# STUDY OF THE MEDIAN AND ULNAR NERVOUS BRANKS TO KAPLAN’S LINE

**DOI:** 10.1590/1413-785220233104e265467

**Published:** 2023-07-31

**Authors:** VICTOR NEY NUNES TOZELLO, TULIO STEFANIN VOLPIANI, VITOR LUIZ MANSUR SILVA, SERGIO APARECIDO DO AMARAL, LUIZ ANGELO VIEIRA, EDIE BENEDITO CAETANO

**Affiliations:** 1 Faculdade de Ciências Médicas e da Saúde, Pontifícia Universidade Católica de São Paulo (PUC), São Paulo, Brazil.; 2 Faculdade de Ciências Médicas e da Saúde, Department of Surgery, Pontifícia Universidade Católica de São Paulo (PUC), São Paulo, Brazil.

**Keywords:** Median Nerve, Ulnar Nerve, Nerve Transfer, Hand, Nervo Mediano, Nervo Ulnar, Transferência de Nervo, Mãos

## Abstract

**Objective:**

This study aims to present lines A1 and A2 in association with Kaplan’s cardinal line (LCK), and relate them to the thenar motor branch of the median nerve (RMTNM) and to the deep branch of the ulnar nerve (RPNU).

**Methods:**

Ten hands of five adult cadavers were dissected.

**Results:**

The RMTNM origin was positioned proximal to the LCK in all limbs. In two, the RMTNM was positioned exactly on the A1 line; in seven, it was on the ulnar side in relation to A1. In one, it was on the radial side relative to the A1. The origin of the RPNU was identified between the pisiform and the LCK in nine limbs; in one, the RPNU was positioned from the ulnar nerve in relation to A2; and in two, the A2 passed exactly at the point of division of the ulnar nerve into superficial branches and deep. We did not identify the positioning of the RPNU on the radial side of the A2 line.

**Conclusion:**

The impact of this study was to identify the anatomical trajectory of these nerves by detaching A1 and A2 along with the KCL, avoiding iatrogenic lesions during surgical procedures. ***Level of Evidence IV, Case Series.***

## RESUMO

**Objetivo:**

Apresentar as linhas A1 e A2 em associação com a linha cardinal de Kaplan (LCK) e relacioná-las ao ramo motor tenar do nervo mediano (RMTNM) e ao ramo profundo do nervo ulnar (RPNU).

**Métodos:**

Foram dissecadas dez mãos de 5 cadáveres adultos.

**Resultados:**

Em todos os membros, a origem do RMTNM posicionou proximal a LCK. Em dois, o RMTNM foi posicionado exatamente na linha A1, em sete foi no lado ulnar em relação à A1. Em um, foi no lado radial em relação à A1. A origem do RPNU foi identificada entre o pisiforme e o LCK em 9 membros, em um, o RPNU foi posicionado a partir do nervo ulnar em relação à A2, em dois, a A2 passou exatamente no ponto de divisão do nervo ulnar em ramos superficial e profundo. Não identificamos o posicionamento do RPNU no lado radial da linha A2.

**Conclusão:**

O impacto deste trabalho é que, ao destacar A1 e A2 juntamente com o LCK, conseguimos identificar a trajetória anatômica desses nervos e, evitar lesões iatrogênicas durante os procedimentos cirúrgicos. **Nível de Evidência IV; Série de Casos.**

## INTRODUCTION

There are reference lines on the palmar surface of the hand, which are used to help locate deep structures. The thenar motor branch of the median nerve (TMBMN) and the deep branch of the ulnar nerve (DBUN) are deep structures that can be injured when performing surgical procedures. The TMBMN is responsible for innervating the muscles in the thenar region that provide the thumb opposition, which is the most important function of the hand. All other intrinsic muscles of the hand are innervated by the DBUN.

**Figure 1:**
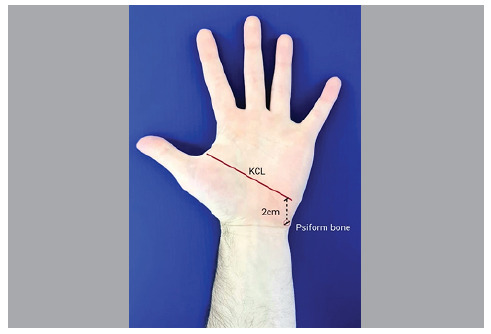
Kaplan’s cardinal line.

In 1953 Kaplan^1^ described a line starting at the apex of the interdigital fold between the thumb and index finger towards the ulnar side of the hand, parallel to the middle palmar fold and called it the cardinal line, which allows establishing the relationship with deep structures such as vessels and nerves of the hand. In 1968, Kaplan himself started to consider the cardinal line as being drawn from the junction of the line which starts at the apex of the interdigital fold between the thumb and index finger, following in the direction of the ulnar border of the hand to a point 2 cm distal to the pisiform bone^2^ (Figure 1). The KCL has often been used as a reference for surgical incisions and to identify deep structures, guide surgical incisions and prevent injuries^2^ ^)-(^ ^5^. The intersection of the KCL with a line following the radial border of the middle finger has been used to locate TMBMN^1^ ^),(^ ^3^ ^)-(^ ^5^. This point of intersection has been described as the location of the origin of the nerve (TMBMN)^3^ ^)-(^ ^5^, or the site where the nerve enters the thenar muscle mass^1^. The intersection of the KCL with a line that follows the ulnar border of the ring finger has been used to locate the annulus of the hamate and the DBUN^1^ ^),(^ ^6^. In addition, the path of the KCL has been used to identify the deep branch of the ulnar nerve^1^, the superficial palmar arch,^1^ ^),(^ ^3^ ^),(^ ^5^ and the distal margin of the transverse carpal ligament^4^. Other investigators have used the KCL to describe the location of surgical incisions for procedures such as open carpal tunnel release^4^ ^),(^ ^5^ ^),(^ ^7^, endoscopic carpal tunnel release^7^ and Dupuytren’s fasciectomy^8^.

KCL = Kaplan's cardinal line.

The aim of this study is to introduce the new lines A1 and A2 in association with the Kaplan’s cardinal line (KCL) and relate those to the thenar motor branch of median nerve (TMBMN) and deep branch of the ulnar nerve (DBUN). By highlighting these new lines along with KCL, we are able to identify the anatomical path of these nerves and furthermore orient surgeons during medical procedures.

## MATERIAL AND METHODS

**Figure 2:**
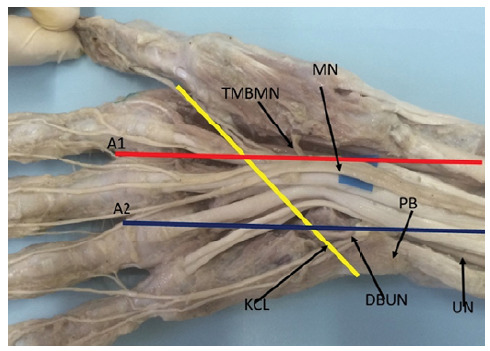
Kaplan’s cardinal line passing directly over the Thenar Motor Branch of the Median Nerve.

**Figure 3:**
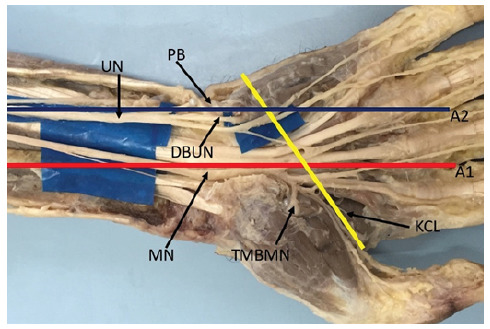
Kaplan's Cardinal Line positioning itself on the ulnar side in relation to the Thenar Motor Branch of the Median Nerve.

**Figure 4:**
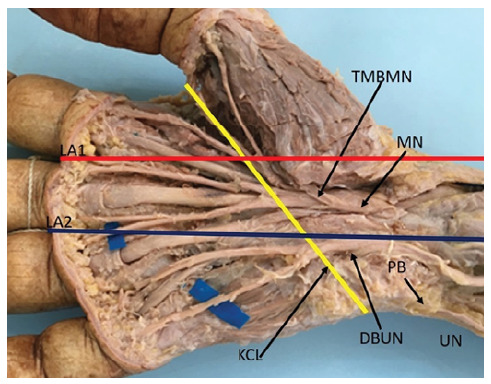
Kaplan's Cardinal Line positioning itself on the radial side in relation to the Thenar Motor Branch of the Median Nerve.

We dissected 10 hands from 5 adult male cadavers, aged 27 to 66 years old, available at the Anatomy Department of PUC-Sorocaba. The dissected hands had no lesions, deformities or scars. The dissections were performed with the aid of a magnifying glass (magnification of 2.5X). The dissection technique was started by an incision proximal to the wrist crease, in the interval between the flexor carpi radialis and palmaris longus muscles, extending distally in the palm of the hand. The median nerve was identified proximally to the transverse carpal ligament, the ligament was sectioned longitudinally on its ulnar side, and its branches were dissected distally. The ulnar nerve was also identified in the wrist, proximal to Guyon’s canal, its deep motor branch was followed distally. Line A1 was drawn from the second interdigital commissure, in a proximal direction following the axis of the hand, which corresponds to the line drawn from the radial border of the middle finger. Similarly, line A2 was drawn from the third commissure, following the axis of the hand and parallel to line A1. Lines A1 and A2 cross the KCL (Figures 2, 3 and 4). The distance between the TMBMN and the DBUN was measured with the KCL. Schematic drawings of the parts were made and systematically photographed. All available specimens adhered to the ethical principles of the institution and the project was evaluated by the Ethics in Research Committee and registered in the Plataforma Brasil, under CAAE No. 14643419.5.0000.5373.

DBUN = deep branch of the ulnar nerve. MN = median nerve. PB = psiform bone. TMBMN = thenar motor branch of median nerve. UN = ulnar nerve.

DBUN = deep branch of the ulnar nerve. KCL = Kaplan's cardinal line MN = median nerve. PB = psiform bone. TMBMN = thenar motor branch of median nerve. UN = ulnar nerve.

DBUN = deep branch of the ulnar nerve. KCL = Kaplan's cardinal line MN = median nerve. PB = psiform bone. TMBMN = thenar motor branch of median nerve. UN = ulnar nerve.

## RESULTS 

We identified that the origin of the TMBMN positioned proximal to the KLC with a distance ranging from 0.3 to 2.5, mean 1.4 cm. In 2 limbs, the TMBMN was positioned exactly on line A1 (Figure 2), in 7 limbs, the TMBMN was positioned on the ulnar side in relation to line A1 (Figure 3) with a distance ranging from 0.2 to 0.6 cm, mean 0.4 cm. In one limb it was positioned 0.3cm from the radial side in relation to line A1 (Figure 4). In all limbs the TMBMN originated from the antero-radial aspect of the median nerve.

The origin of the DBUN, was identified between the psiphorme bone and the KCL in 9 limbs. With distance ranging from 0.4 to 0.9, with an average of 0.7 cm proximal to the KCL. In 1 limb the DBUN originated 2.5 cm proximal to the piriform bone (Figure 3). We did not identify the DBUN originating distal to the KCL. In 8 limbs the UPRNB was positioned on the ulnar side in relation to line A2, (Figure 2), in 2 limbs the line A2 passed exactly at the point of division of the ulnar nerve into superficial and deep branches (Figure 4). We did not identify the DBUN positioned on the radial side of line A2.

## DISCUSSION 

Analyzing the literature, we observed that there is no consensus regarding the definition of KCL, four different descriptions were found^1^ ^),(^ ^2^ ^),(^ ^9^ ^),(^ ^10^. Vella et al^9^ reported that their research showed that most surgeons who participated in their research used KCL as a reference in the surgical act. In the present study, we considered the KCL definition of^9^, i.e. the trace of the junction starting at the apex of the interdigital fold between the thumb and index finger, following towards the ulnar border of the hand, up to a point 2 cm distal to the pisiform bone^2^. 

Kaplan’s cardinal line has been used as a surface marker in several clinical and anatomical studies. In this study, we identified that the origin of the TMBMN was positioned proximal to the KCL with a distance ranging from 0.3 to 2.5 averaging 1.4 cm. In 2 limbs, the line A1 passed exactly over the TMBMN, in 7 it was positioned on the ulnar side in relation to the line A1 with distance varying from 0,2 to 0,6 cm, mean of 0,4 cm, in another limb it was positioned 0,3 cm on the radial side in relation to the line A1 (Figure 4). In all limbs the TMBMN originated from the antero-radial aspect of the median nerve.

Eskandari et al^10^, performed a study on 37 hands of 34 patients undergoing carpal tunnel release procedure. A radiological marking technique was used to determine the location of the TMBMN, in relation to the KCL and also in relation to the line accompanying the radial margin of the middle finger, which corresponds to line A1 in our study. They concluded that the RMT had a mean ulnar displacement of 12.6 mm (range 4.0 to 19.7 mm) from the radial lateral line of the middle finger and was located 4.4 mm (range 0 to 9.5 mm) proximal to the cardinal line. Our findings agree with those of Eskandari et al^10^, regarding the KCL because in all limbs the TMBMN was positioned proximal to the KCL. In relation to the radial-ulnar impingement, we registered slightly different results. According to Eskandari el al^10^, in all cases the TMBMN was positioned on the ulnar side in relation to the line following the radial margin of the middle finger. In this study we identified in 7 limbs, the TMBMN was positioned on the ulnar side in relation to line A1, agreeing with these authors. In another limb it was positioned on the radial side (Figure 3), in two limbs the line A1 passed exactly over the TMBMN.

The origin of the DBUN was identified between the psiform and the KCL in 9 limbs. With distance ranging from 0.4 to 0.9, average of 0.7 cm proximal to the KCL. In 1 limb the DBUN originated 2,5 cm proximal to the piriformis. We did not identify the DBUN originating distal to the KCL. In 8 limbs the DBUN was positioned from the ulnar lobe in relation to line A2, in 2 limbs the line A2 passed exactly in the point of division of the ulnar nerve in superficial and deep branches. We did not identify the DBUN positioned on the radial side of line A2. 

We did not find in the literature any work that directly relates the DBUN to the KCL. Bini and Leclercq^11^ studied the DBUN in 21 hands of recently deceased cadavers, with the purpose of analyzing its branches to the intrinsic muscles of the hand. They used three anatomical points as reference: the biestiloid line, the radial flexor tendon of the carpus, and the pisiform bone; they did not inform why they did not also use the KCL as reference. Dashe and Jones^12^ presented a method for safe exposure and removal of the hamate annulus in cases of pseudoarthrosis with pain symptoms. They used the KCL and the line accompanying the ulnar margin of the ring finger as a reference for the access route, to avoid damage to the DBUN. Choi and Yoon^13^ evaluated the DBUN in 60 wrists of 30 healthy adult patients using high-resolution ultrasonography. The course of the RMNU was evaluated using the hamate annulus and skin depth as reference. They did not report why Kaplan’s line was not used as a reference.

Some authors have related the KCL to the arterial arches of the palmar surface of the hand. Panchal and Trzeciak^14^ performed an anatomical study in 30 cadavers, dissecting 60 hands, to describe the relationship between Kaplan’s cardinal line and the superficial palmar arterial arch. They stated that from a clinical point of view, Kaplan’s cardinal line is the most predictable marker to identify the superficial palmar arch. McLean et al^15^ performed an anatomical study on 48 cadaveric hands in specimens between 50 and 75 years old, with the purpose of assessing the distance of the superficial palmar arch and the KCL. Similarly, Anand and Trzeciak^16^ anatomically correlated the relationship of Kaplan’s cardinal line with the superficial and deep palmar arterial arches. Kwiatkowska et al^17^ dissected 20 upper limbs from cadavers. They related the deep palm structures to the palmar folds, and concluded that the palmar folds vary considerably between people and that genetics has an influence on the formation of the folds. They considered that the middle palmar crease was parallel to the KCL. 

We are aware of the limitations in the present study, such as the limited number of cases and the non-living tissue conditions. Although we could not examine in vivo conditions, cadaver preparation does not alter the positioning of the anatomical structures. The highlight of this work is that we found no anatomical studies in the literature that relate the KCL to the TMBMN and DBUN.

## CONCLUSION

In this study we propose new reference lines, named A1 and A2, to guide hand surgeries. In all members the TMBMN and DBUN were positioned close to the KCL. The TMBMN was positioned on the ulnar side in relation to the A1 line in 7 limbs; on one of the radial side; in two passed over the TMBMN. The DBUN was positioned on the ulnar side in relation to the A2 line, between the psiform bone and the KCL in 9 limbs in 1 proximal to the psiform bone. The impact of this work is that by highlighting lines A1 and A2 together with the KCL, we are able to identify the anatomic trajectory of these nerves and consequently avoid iatrogenic injures during surgical procedures. 
